# Effects of High Phosphorus Diet on Bone Metabolism-Related Gene Expression in Young and Aged Mice

**DOI:** 10.1155/2014/575932

**Published:** 2014-11-19

**Authors:** Shinichi Katsumata, Hiroshi Matsuzaki, Rie Katsumata-Tsuboi, Mariko Uehara, Kazuharu Suzuki

**Affiliations:** ^1^Department of Nutritional Science, Faculty of Applied Bioscience, Tokyo University of Agriculture, 1-1-1 Sakuragaoka, Setagaya-ku, Tokyo 156-8502, Japan; ^2^Department of Nutritional Science and Food Safety, Faculty of Applied Bioscience, Tokyo University of Agriculture, 1-1-1 Sakuragaoka, Setagaya-ku, Tokyo 156-8502, Japan

## Abstract

In this study, the effects of high phosphorus (P) diet on bone metabolism-related gene expression were investigated in young and aged mice. Twelve- and 80-week-old ddY male mice were divided into two groups, respectively, and fed a control diet containing 0.3% P or a high P diet containing 1.2% P. After 4 weeks of treatment, serum parathyroid hormone (PTH) concentration was significantly higher in the high P groups than in the control groups in both young and aged mice and was significantly higher in aged mice than in young mice fed the high P diet. High P diet significantly increased receptor activator of NF-*κ*B ligand (RANKL) mRNA in the femur of both young and aged mice and significantly increased the RANKL/osteoprotegerin (OPG) mRNA ratio only in aged mice. High P diet significantly increased mRNA expression of transient receptor potential vanilloid type 6, calbindin-D9k, and plasma membrane Ca^2+^-ATPase 1b in the duodenum of both young and aged mice. These results suggest that high P diet increased RANKL mRNA expression in the femur and calcium absorption-related gene expression in the duodenum regardless of age. Furthermore, the high P diet-induced increase in PTH secretion might increase the RANKL/OPG mRNA ratio in aged mice.

## 1. Introduction

High phosphorus (P) intake is known as one of the risk factors for impaired bone health. Several researchers have investigated the adverse effects of a high P diet on bone metabolism. In human adults, a diet containing P additives increases urinary hydroxyproline excretion, a bone resorption marker [1]. In growing male rats, high P intake decreases bone mineral density (BMD) and bone strength [2]. Elevation in parathyroid hormone (PTH) secretion is considered one of the mechanisms by which a high P diet impairs bone metabolism [3].

Bone metabolism results from the balance between osteoblastic bone formation and osteoclastic bone resorption. Chronic PTH stimulation is known to induce osteoclastogenesis. PTH stimulates osteoblasts, which produce mediators of osteoclastic bone resorption such as macrophage colony-stimulating factor (M-CSF), interleukin-6 (IL-6), or receptor activator of NF-*κ*B ligand (RANKL) [4–6]. We previously reported that a high P diet increased RANKL mRNA expression and the osteoclast number in rats [3]. It could therefore be deduced that high P diet-induced elevated PTH secretion leads to an increase in RANKL expression, which enhances osteoclastic bone resorption.

Aging is also one of the risk factors of bone loss in elderly individuals. Overton and Basu suggested that bone loss occurs with increasing age at a rate of approximately 1% per year averaged over the age range of 29–76 y [7]. In a previous mouse study by Ferguson et al., bone mass and mechanical properties approached mature levels by 12 weeks of age while age-related osteopenia was observed after 42 weeks of age [8]. We hypothesized that a high P diet would accelerate age-related bone loss. We previously reported the effects of a high P diet on mechanical properties of the femur in 4-, 12-, 24-, and 80-week-old mice [9]. The results showed that a high P diet decreased the breaking force of the femur in 80-week-old mice and the stiffness of the femur in 24- and 80-week-old mice. We also found that a high P diet increased serum PTH concentration in 12-, 24-, and 80-week-old mice, and 80-week-old mice had a higher serum PTH concentration than mice at other ages. Therefore, it was thought that a high P diet strongly influences aged mice in terms of PTH response. However, the mechanisms are not fully understood.

The purpose of this study was to clarify the mechanism by which a high P diet affects bone metabolism in older mice. We assessed the changes in bone metabolism in young and aged mice fed a high P diet by measuring the mRNA expression of bone metabolism mediators using real-time polymerase chain reaction (PCR).

## 2. Methods

### 2.1. Experimental Design

This study was approved by the Tokyo University of Agriculture Animal Use Committee. The mice were maintained in accordance with the university guidelines for the care and use of laboratory animals. The experimental diets were based on the AIN-93G diet (Table 1) [10]. A control diet containing 0.3% P and a high P diet containing 1.2% P were prepared. Each experimental diet contained 0.5% calcium (Ca). Twenty-four 10-week-old ddY male mice were purchased from SLC (Shizuoka, Japan) and housed individually in stainless cages in a room maintained at 22°C with a 12-hour light/dark cycle. Half of the mice were fed a commercial diet (CE-2, CLEA Japan, Tokyo, Japan) until 78 weeks of age. All mice were fed the control diet for 2 weeks of acclimatization period. After the acclimatization period, 12 young (12-week-old) and 12 aged (80-week-old) mice were randomly divided into two experimental groups and fed the control diet or the high P diet for 4 weeks. They were given free access to the diets and distilled water. Their urine samples were collected during the 5 days prior to euthanasia for the further analyses. At the end of the experimental period, all mice were euthanized under anesthesia and blood, bone, and duodenum samples were collected for analyses. The blood samples were centrifuged and the supernatants were used as serum samples. The femur samples were removed and cleaned of all soft tissues. All samples were stored at –80°C until further analyses.

### 2.2. Serum and Urine Analyses

The serum Ca level was analyzed by atomic absorption spectrophotometry (Hitachi A-2000; Hitachi, Tokyo, Japan) according to the method of Gimblet et al. [11]. The serum P level was analyzed by colorimetry using Phospha *C*-test Wako (Wako Pure Chemical Industries, Osaka, Japan). Serum PTH concentration was measured using the mouse intact PTH enzyme-linked immunosorbent assay (ELISA) kit (ALPCO, NH, USA). Serum intact osteocalcin (OC) concentration was measured using the mouse osteocalcin EIA kit (Biomedical Technologies, MA, USA). Urinary C-terminal telopeptide of type I collagen (CTx) level was measured using RatLaps EIA kit (Immunodiagnostic Systems, Boldon, UK). Urinary creatinine level was measured using the Jaffe reaction, as described by Lustgarten and Wenk [12]. Urinary CTx level was normalized to the urinary creatinine level.

### 2.3. Isolation of Total RNA and Real-Time PCR

Total RNA was isolated from the homogenized femurs or duodenum by using TRIzol reagent (Life Technologies, CA, USA) according to the manufacturer's specifications. The amount and purity of the RNA were assessed using a NanoDrop 2000c (Thermo Fisher Scientific, MA, USA). Complementary DNA (cDNA) was synthesized using the High-Capacity RNA-to-cDNA Kit (Applied Biosystems, CA, USA). For real-time PCR, the reaction mixture was prepared using the TaqMan Gene Expression Master Mix (Applied Biosystems) with TaqMan gene expression assays (Applied Biosystems) for mouse PTH receptor (Assay ID: Mm00441046_m1), mouse RANKL (Assay ID: Mm00441906_m1), mouse osteoprotegerin (OPG) (Assay ID: Mm01205928_m1), mouse tartrate resistant acid phosphatase (TRAP) (Assay ID: Mm00475698_m1), mouse runt related transcription factor 2 (Runx2) (Assay ID: Mm00501580_m1), mouse Osterix (Assay ID: Mm04209856_m1), mouse alkaline phosphatase (ALP) (Assay ID: Mm00475834_m1), mouse osteopontin (OPN) (Assay ID: Mm00436767_m1), mouse OC (Assay ID: Mm03413826_mH), mouse type I collagen (Col1a1) (Assay ID: Mm00801666_g1), mouse transient receptor potential vanilloid type 6 (TRPV6) (Assay ID: Mm00499069_m1), mouse calbindin-D9k (Assay ID: Mm00486654_m1), mouse plasma membrane Ca^2+^-ATPase 1b (PMCA1b) (Assay ID: Mm01245805_m1), and mouse glyceraldehyde-3-phosphate dehydrogenase (GAPDH) (Assay ID: Mm99999915_g1). Real-time PCR was performed using a StepOne Real-Time PCR System (Applied Biosystems). The mRNA expression was normalized to GAPDH mRNA as a housekeeping gene. The value of the young mice fed the control diet was considered to be 1.00.

### 2.4. Statistical Analysis

Results are expressed as the mean ± SEM for each group of six mice. After two-way analysis of variance (ANOVA), Fisher's protected least significant difference (PLSD) test was used to determine significant differences among the groups. The homogeneity of variance was analyzed with Levene's test. Differences were considered to be significant when the *P* value was less than 0.05.

## 3. Results

### 3.1. Body Weight

In both young and aged mice, there was no significant difference in the initial body weight between mice fed the control and high P diets (Table 2). The initial body weight of aged mice was significantly higher than that of young mice. There was no significant difference in the final body weight among groups.

### 3.2. Serum Ca, P, and PTH Concentrations

There were no significant differences in serum Ca and P concentrations among the groups (Table 2). In both young and aged mice, the high P diet significantly increased serum PTH concentration. Although there was no significant difference in serum PTH concentration between young and aged mice fed the control diet, serum PTH concentration was significantly higher in aged mice than in young mice fed the high P diet.

### 3.3. Markers of Bone Turnover

In both young and aged mice, the high P diet significantly increased serum OC concentration (Table 2). In mice fed both the control and high P diets, serum OC concentration was significantly lower in aged mice than in young mice. In both young and aged mice, the high P diet significantly increased urinary excretion of CTx. Although there was no significant difference in urinary excretion of CTx between young and aged mice fed the control diet, urinary excretion of CTx was significantly lower in aged mice than in young mice fed the high P diet.

### 3.4. mRNA Expression in the Femur

**Figure 1:**
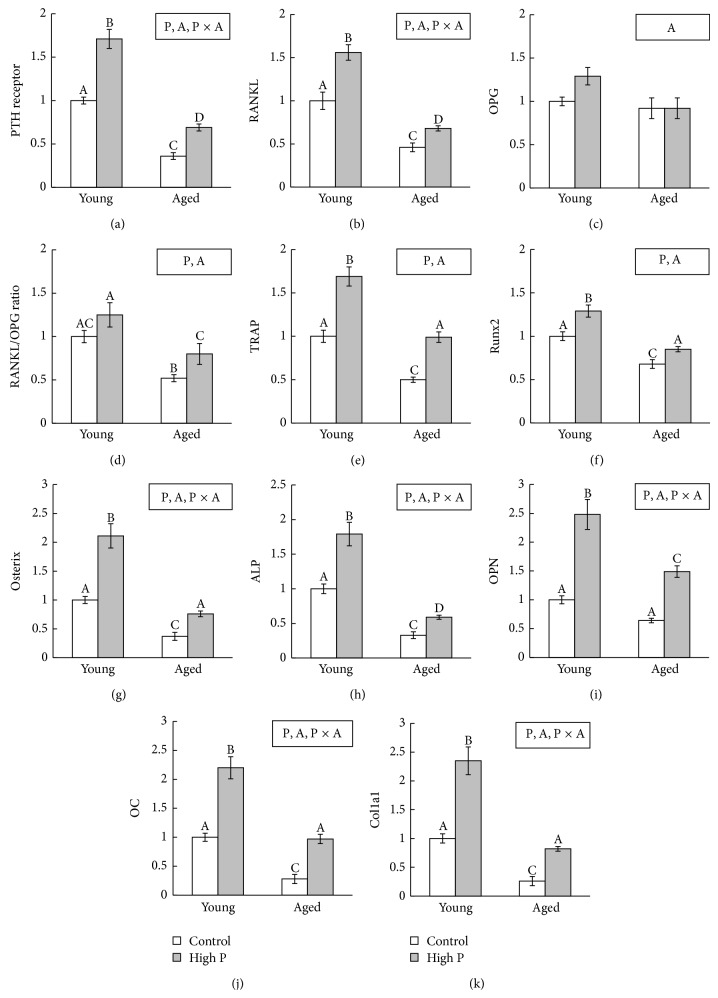
Bone metabolism-related gene expression in the femur. (a) PTH receptor; (b) RANKL; (c) OPG; (d) RANKL/OPG ratio; (e) TRAP; (f) Runx2; (g) Osterix; (h) ALP; (i) OPN; (j) OC; (k) Col1a1. The data indicate the mean ± SEM of 6 mice. ^A, B, C, D^The different letters denote significant differences (*P* < 0.05). Significant effect (*P* < 0.05): P = effect of high P diet; A = effect of age; P × A = effect of interaction. The value of young mice fed a control diet is considered to be 1.00.

In both young and aged mice, the high P diet significantly increased mRNA expression of PTH receptor, RANKL, TRAP, Runx2, Osterix, ALP, OPN, OC, and Col1a1 (Figure 1) compared to the control diet. In mice fed the control and high P diets, mRNA expression of PTH receptor, RANKL, TRAP, Runx2, Osterix, ALP, OPN, OC, and Col1a1 was significantly lower in aged mice than in young mice. There was no significant difference in OPG mRNA expression among the groups. The high P diet significantly increased RANKL/OPG ratio in aged mice but did not in young mice.

### 3.5. mRNA Expression in the Duodenum

**Figure 2:**
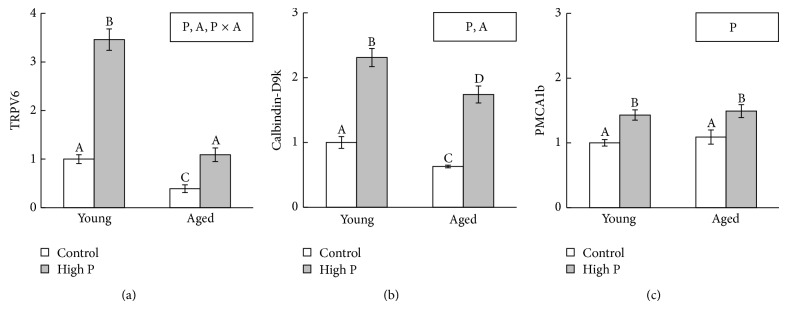
Ca absorption-related gene expression in the duodenum. (a) TRPV6; (b) calbindin-D9k; (c) PMCA1b. The data indicate the mean ± SEM of 6 mice. ^A, B, C, D^The different letters denote significant differences (*P* < 0.05). Significant effect (*P* < 0.05): P = effect of high P diet; A = effect of age; P × A = effect of interaction. The value of young mice fed a control diet is considered to be 1.00.

In both young and aged mice, high P diet significantly increased mRNA expression of TRPV6, CaBP9k, and PMCA1b (Figure 2) compared to the control diet. In mice fed the control and high P diets, mRNA expression of TRPV6 and CaBP9k was significantly lower in aged mice than in young mice.

## 4. Discussion

In humans, bone loss occurs with increasing age at a rate of approximately 1% per year averaged over the ages of 29–76 years [7]. Therefore, aging is one of the risk factors for bone loss. In a previous mouse study, bone mass and mechanical properties were shown to approach mature levels by 12 weeks of age, and age-related osteopenia was observed after 42 weeks [8]. In this study, we investigated bone metabolism by measuring markers of bone formation and resorption, serum OC concentration [13], and urinary CTx excretion [14]. Serum OC concentration was significantly lower in aged mice than in young mice fed the control diet, whereas there is no difference in urinary excretion of CTx between young and aged mice fed the control diet. These results showed that aged mice present a decrease in bone formation, and it appears that the balance between bone formation and resorption may be disrupted in aged mice.

Bone formation is mediated by osteoblasts. Using gene-deficient mouse models, Runx2 and Osterix were shown to be essential transcription factors for osteoblast differentiation and bone formation [15, 16]. In this study, Runx2 and Osterix mRNA expression were significantly lower in aged mice than in young mice. These results suggest that aging leads to a reduction in osteoblast differentiation and that Runx2 and Osterix mRNA expression changes are associated with a decrease in bone formation in aged mice. Consequently, decreases in mRNA expression of ALP and bone matrix proteins such as OPN, OC, and Col1a1 also occurred in aged mice. Ikeda et al. showed that the mRNA expression of OPN, OC, and Col1a1 decreased in both cortical and trabecular bones in aged rats compared to young animals [17]. In addition, Cao et al. reported that ALP and Col1a1 expression declined in aged mice compared to young mice [18]. Thus, aging results in a decrease in bone formation with declined osteoblast-related gene expression. With regard to bone resorption, this study showed that RANKL and TRAP mRNA expression were decreased in aged mice compared to young mice, despite unchanging serum PTH concentration. Since PTH stimulates RANKL [6], the result of RANKL mRNA expression seems to contradict that of serum PTH concentration. However, PTH receptor mRNA expression was decreased in aged mice compared to young mice in this study. This result suggested that PTH action was suppressed, which decreased RANKL mRNA expression in aged mice. Though urinary excretion of CTx was unchanged, expression of bone resorption-rerated genes was decreased in aged mice compared to young mice. However, it is generally known that age-related increase in serum PTH contributes to the increase in bone resorption [19]. Therefore, results of serum PTH concentration and bone resorption between young and aged mice in this study are contradictory. Further studies with the increase in number of mice per group are needed to address these discrepancies.

A decline in intestinal Ca absorption is also one of the causes of age-related bone loss. The transfer of Ca via the intestine occurs through both transcellular and paracellular pathways [20]. The transcellular Ca pathway, which is affected by 1,25-dihydroxyvitamin D (1,25(OH)_2_D), has been proposed to involve Ca entry via TRPV6, intracellular diffusion of Ca by calbindin-D9k, and basolateral extrusion of Ca by PMCA1b [21]. In this study, TRPV6 and calbindin-D9k mRNA expression in the duodenum were decreased in aged mice compared to young mice. Wood et al. demonstrated that plasma 1,25(OH)_2_D, duodenal calbindin D protein, and Ca absorption decreased with age in rats [22]. From this study, we could deduce that similar findings would be found in aged mice. Therefore, a decrease in serum 1,25(OH)_2_D might reflect our results on TRPV6 and calbindin-D9k mRNA expression.

Many studies have reported that high P intake induces an increase in serum PTH concentration in humans [1, 23] and animals [2, 3, 24]. In this study, the high P diet increased serum PTH concentration in both young and aged mice and was greater in aged mice than in young mice fed the high P diet. Similar to our previous study [9], these results suggest that the response to a high P diet in terms of PTH secretion might be different and greater with aging. Kidney function decreases with age [25], and declining kidney function causes an increase in PTH secretion [26]. Furthermore, we previously reported that high P diet decreased kidney function in rats [27]. Therefore, the combination of aging and high P diet might be one of the reasons that higher serum PTH concentration was observed in aged mice fed the high P diet.

We previously reported that a high P diet increased RANKL mRNA expression and bone resorption in growing rats [3]. RANKL is a mediator of osteoclastic bone resorption and is stimulated by PTH [6]. Therefore, it was suggested that elevated PTH secretion induced by the high P diet led to an increase in RANKL expression, which increased bone resorption. In this study, the high P diet significantly increased urinary excretion of CTx in both young and aged mice. Regarding mRNA expression of bone resorption-related molecules in the femora, the high P diet significantly increased RANKL and TRAP mRNA expressions in both young and aged mice. These results suggested that the high P diet enhanced bone resorption independently of age. RANKL actions are inhibited by OPG, which acts as a decoy receptor by blocking RANKL binding to its receptor [28]. In this study, the high P diet significantly increased the RANKL/OPG ratio in aged mice, whereas the ratio was unchanged in young mice. Many reports have supported the assertion that the increase in RANKL/OPG ratio promotes osteoclastogenesis, accelerates bone resorption, and induces bone loss [29]. Our previous study showed that a high P diet decreased the breaking force and stiffness of the femur in aged mice compared to young mice [9]. Our findings show that the high P diet in the aged mice leads to increased PTH secretion and consequent increases in the RANKL/OPG ratio accelerating osteoclastogenesis.

Previous studies showed that PTH regulates Runx2 and Osterix mRNA expression [30, 31]. In this study, the high P diet significantly increased serum OC concentration and mRNA expression of Runx2, Osterix, ALP, OPN, OC, and Col1a1 in both young and aged mice. From the results of bone resorption and formation markers, high bone turnover with resorption exceeding formation was observed. Since high bone turnover is a risk factor for bone fracture and osteoporosis [32], a high P diet might be a risk factor for bone loss not only in young mice but also in aged mice.

PTH secretion might reflect a decrease in Ca absorption in the high P diet group. Although the mechanism underlying the high P diet-induced decreased Ca absorption remains unknown, it is thought that the formation of insoluble Ca and P salts in the intestinal lumen is an important factor [33]. Our previous study also showed that high P diet decreased Ca absorption in female rats [34]. While we did not evaluate Ca absorption in this study, it is possible to estimate the decrease in Ca absorption by high P diet in young and aged mice. However, this study showed that the high P diet significantly increased mRNA expression of TRPV6, CaBP9k, and PMCA1b in both young and aged mice. Previous study demonstrated that Ca restriction during lactation stimulated Ca-binding protein and active Ca transport in jejunum and ileum [35]. Furthermore, Ca deficient diet resulted in an increase in duodenal PMCA mRNA in chickens [36]. These studies suggested that 1,25(OH)_2_D-regulated Ca transporters might be stimulated by low Ca status in the intestinal lumen. Thus, a decrease in soluble Ca induced by high P diet might lead to TRPV6, CaBP9k, and PMCA1b mRNA expression, and these increases in 1,25(OH)_2_D-regulated gene expression seem to compensate for a decrease in Ca absorption by the high P diet. In brief, our results suggest that high P diet accelerates the transcellular Ca pathway, though absorbed amount of Ca was insufficient to maintain serum PTH concentration. It is known that fibroblast growth factor 23 (FGF23) and 1,25(OH)_2_D as well as PTH are key factors for Ca and P metabolism. FGF23 inhibits P reabsorption and 1,25(OH)_2_D synthesis in the kidney [37]. Furthermore, high P diet increased serum FGF23 concentration in mice [38]. Therefore, measuring serum FGF23 and 1,25(OH)_2_D is important to fully elucidate the mechanisms by which high P diet changes Ca and P metabolism, and further studies are needed to clarify the details.

## 5. Conclusion

In conclusion, we demonstrated that the high P diet increased bone metabolism-related gene expression in both young and aged mice. Furthermore, the high P diet affected PTH secretion differently in young and aged mice, leading to an increase in the RANKL/OPG mRNA ratio in aged mice.

## Figures and Tables

**Table 1:** Composition of the experimental diets.

	Control	High P
	g/kg diet	
Casein	200.0	200.0
Corn starch	529.486	489.938
Sucrose	100.0	100.0
Soybean oil	70.0	70.0
Cellulose powder	50.0	50.0
AIN-93G mineral mixture	35.0	35.0
AIN-93 vitamin mixture	10.0	10.0
L-Cysteine	3.0	3.0
Choline bitartrate	2.5	2.5
*tert*-Butylhydroquinone	0.014	0.014
KH_2_PO_4_	—	39.548

**Table 2:** Body weight, serum Ca and P, serum PTH, and markers of bone turnover.

	Young		Aged		Two-way ANOVA^1^
	Control	High P	Control	High P	
Initial body weight (g)	44.10 ± 0.79^A^	44.20 ± 0.65^A^	52.71 ± 2.17^B^	53.84 ± 2.56^B^	A
Final body weight (g)	48.66 ± 1.43	46.18 ± 0.67	51.47 ± 2.28	50.69 ± 2.19	
Serum Ca (mg/dL)	9.15 ± 0.17	8.67 ± 0.20	8.81 ± 0.27	8.60 ± 0.12	
Serum P (mg/dL)	9.23 ± 0.41	9.49 ± 0.47	9.07 ± 0.68	9.08 ± 0.60	
Serum PTH (pg/mL)	52.8 ± 12.5^A^	181.4 ± 33.0^B^	76.7 ± 13.8^A^	306.1 ± 51.1^C^	P, A
Serum OC (ng/mL)	26.58 ± 0.83^A^	37.53 ± 2.94^B^	7.31 ± 0.48^C^	13.44 ± 0.78^D^	P, A
Urine CTx (*μ*g/mmol creatinine)	5.98 ± 0.91^A^	16.73 ± 1.22^B^	5.62 ± 1.02^A^	9.61 ± 1.13^C^	P, A, P × A

The data indicate the mean ± SEM of 6 mice.

^A,B,C,D^The different superscript letters denote significant differences (*P* < 0.05).

^1^Significant effect (*P* < 0.05): P = effect of high P diet; A = effect of age; P × A = effect of interaction.
